# Hemorrhagic and Ecthymatous Varicella in Immunosuppressed Adults: Report of Two Cases

**DOI:** 10.7759/cureus.59409

**Published:** 2024-04-30

**Authors:** Honey Chacko, C Vijay Krishna, Sheela Kuruvila

**Affiliations:** 1 Dermatology, NMC Specialty Hospital, Abu Dhabi, ARE; 2 Dermatology, Thumbay University Hospital, Ajman, ARE; 3 Dermatology, Aarupadai Veedu Medical College, Pondicherry, IND

**Keywords:** treatment of varicella in immunosuppressed, atypical varicella, varicella in immunosuppressed, ecthymatous varicella, hemorrhagic varicella

## Abstract

Varicella infection in immunosuppressed adults can be severe with atypical presentation of skin lesions. Hemorrhagic and ecthymatous varicella is a rare entity and can be misdiagnosed due to its atypical presentation. In its severe form, individuals with underlying cell-mediated immunodeficiency disorders have a high risk of developing multiple organ involvement associated with varicella-zoster virus infection. Here, we report two cases of primary varicella with hemorrhagic and ecthymatous skin lesions in adults receiving systemic immunosuppressive drugs for autoimmune disorders. There are only a few case reports on hemorrhagic and ecthymatous varicella. Hence, this case report highlights the atypical presentation of varicella in immunosuppressed adults, which necessitates an early diagnosis and prompt treatment as a lifesaving step.

## Introduction

Primary varicella, an extremely common communicable disease worldwide caused by varicella-zoster virus (VZV), is mostly a disease of childhood. However, in tropical countries such as India, the incidence of primary varicella is significantly greater in adults [1,2]. The disease is usually self-limiting in immunocompetent children but can be associated with a variety of serious and potentially lethal complications in adults and immunocompromised individuals [3,4].

Primary varicella infection in immunosuppressed can present with atypical skin lesions such as ecthymatous [5], hyperkeratotic, verrucous [6], and hemorrhagic. The skin lesions continue to erupt for a longer duration than normal with increased severity. The impaired cell-mediated immunity [7] is thought to be responsible for the atypical clinical presentation in such patients. Other atypical clinical presentations seen in immunosuppressed individuals, or patients with underlying skin disorders such as burns, eczema, and cutaneous lymphoma are purpura fulminans, necrotizing fascitis, and hemorrhagic or bullous varicella [7]. Complications associated with VZV infection have been observed in immunosuppressed patients, neonates, and to a lesser extent in normal adults. Severe disseminated disease is associated with significant mortality up to 7%-10% [1].

Hemorrhagic varicella is rare and has been seldom reported in the dermatology literature [8]. Here, we report two cases of hemorrhagic and ecthymatous varicella in adults receiving immunosuppressive drugs for autoimmune disorders.

## Case presentation

Patient 1

A 30-year-old woman, receiving oral prednisolone and azathioprine for autoimmune hepatitis diagnosed one month ago, was referred to the dermatology department with complaints of fever and rash of five days duration. The rash initially appeared over the face and upper limbs and later progressed to involve the trunk and lower limbs. As a child, she had never contracted varicella. Recently, she had come in contact with a family member who had active skin lesions of varicella. On examination, she was febrile and icteric, but the rest of her vitals were stable. Abdominal examination showed a tender and enlarged liver. Examination of other organ systems was unremarkable.

**Figure 1:**
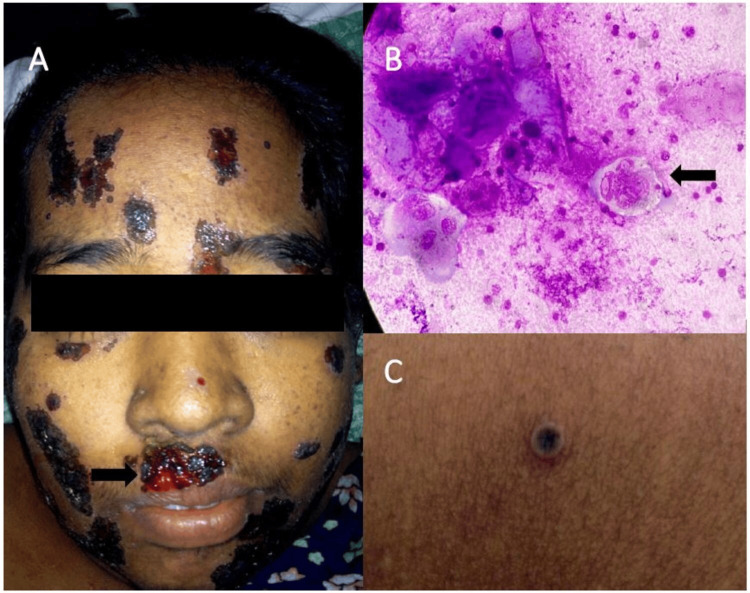
(A) Multiple erosions with heaped up hemorrhagic crusts resembling ecthyma (white arrow). (B) Tzanck smear (100x oil immersion) showing multi-nucleated giant cells with intra-nuclear inclusion bodies (black arrow). (C) Umbilicated vesicle seen over the left forearm.

Vesicles on the face and upper trunk ruptured to form erosions that bled increased in size and were covered by thick adherent hemorrhagic crusts resembling ecthyma (Figure 1A). There was no mucosal involvement. Scalp, palms, and soles were uninvolved. Tzanck smear from a vesicle on the forearm revealed multinucleated giant cells with intranuclear inclusion bodies (Figure 1B). Cutaneous examination revealed multiple umibilicated vesicles containing sero-hemorrhagic fluid on the trunk and extremities (Figure 1C).

Laboratory investigations showed increased total leukocyte count with low hemoglobin levels and platelet count. The direct bilirubin level, SGOT and SGPT were high with decreased serum albumin, and increased prothrombin level. Laboratory investigations are tabulated (Table 1).

**Table 1:** Selected laboratory data of patient 1

VARIABLE	PATIENT VALUES	REFERENCE RANGE
White blood cell count(×10^9^/L)	17.3	3.5-9.5
Neutrophil(×10^9^/L)	90	40-75
Platelets count(×10^9^/L)	72,000	150,000-450,000
Hemoglobin(g/dL)	9.8	13-17
Total Bilirubin(µmol/L)	19.2	1.7-20.5
Direct Bilirubin(µmol/L)	14.4	1.7-5.1
Alanine aminotransferase/SGPT(U/L)	108	5-40
Aspartate aminotransferase/SGOT(U/L)	75	7-40
Serum Albumin(g/dL)	2.3	3.4-5.4
Prothrombin(seconds)	26.5	11-13.5
INR	2.1	0.8-1.1
D- dimer(g/L)	less than 0.50	less than or equal to 0.50
Fibrin degradation products(mcg/mL)	less than 10	less than 10

The patient was admitted for further evaluation. Pus for culture sent from the ecthymatous lesions over the face showed no bacterial growth. Anti-varicella IgM antibodies were positive, however, other viral serologies such as HIV, HBsAg, HCV antibodies, and HSV antibodies were negative. 

On the basis of the clinical picture and laboratory tests, a diagnosis of hemorrhagic and ecthymatous varicella was made. Under admission, Azathioprine was withheld and intravenous acyclovir 10mg/kg 8 hourly, was started within 24 hours. After three days of instituting treatment, her skin lesions and laboratory parameters showed improvement. Unfortunately, the patient discontinued further treatment against medical advice and was lost to follow-up.

Patient 2

A 22-year-old woman receiving prednisolone and mycophenolate mofetil for lupus nephritis diagnosed two years ago presented to the emergency department with fever, fatigue, and multiple fluid-filled lesions over the scalp, face, trunk, and extremities of one-week duration. She had a history of contact with a person with varicella two weeks prior to her presentation to us.

**Figure 2:**
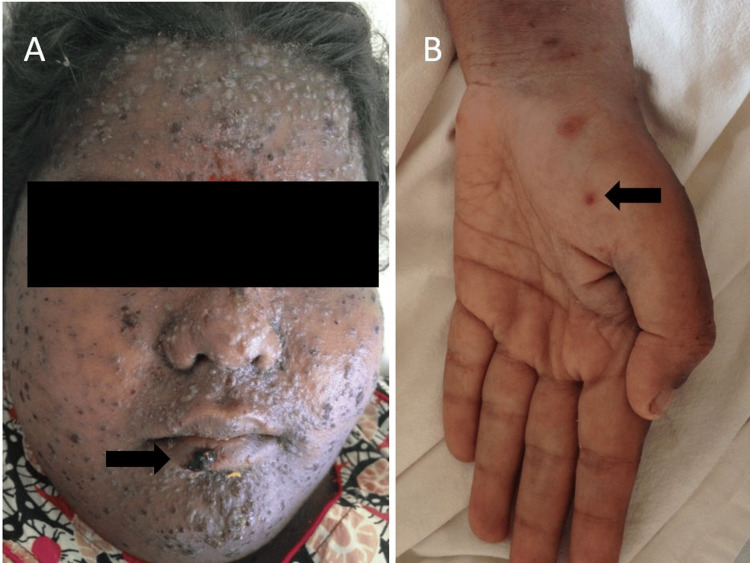
(A) Multiple vesicles, some of them umbilicated, seen over the face. Erosions covered with hemorrhagic crusts are also seen (white arrow). (B) Few scattered umbilicated vesicles containing hemorrhagic fluid over the palms (white arrow).

On examination, she was febrile but other vitals were stable. Systemic examination was within normal limits. In addition, crusted hemorrhagic plaques were seen on the scalp and face (Figure 2A). Cutaneous examination revealed multiple vesicles on scalp and face with scattered umbilicated vesicles containing sero-hemorrhagic fluid on the trunk and extremities including the palms and soles (Figure 2B). Mucosae were uninvolved. Tzanck smear from a vesicle over the forearm revealed multinucleated giant cells.

The patient was admitted and evaluated. Laboratory investigations showed increased total leukocyte count with low hemoglobin levels and platelet counts. The SGOT and SGPT levels were high with normal bilirubin, serum albumin, and prothrombin levels. The fibrin degradation products were raised however there was no evidence of disseminated intravascular coagulation. Laboratory investigations are tabulated (Table 2).

**Table 2:** Selected laboratory data of patient 2

VARIABLE	PATIENT VALUES	REFERENCE RANGE
White blood cell count(×10^9^/L)	22.9	3.5-9.5
Neutrophil(×10^9^/L)	95	40-75
Platelets count(×10^9^/L)	21,000	150,000-450,000
Hemoglobin(g/dL)	10.8	13-17
Total Bilirubin(mg/dl)	0.4	1.7-20.5
Direct Bilirubin(mg/dl)	0.3	1.7-5.1
Alanine aminotransferase/SGPT(U/L)	231	5-40
Aspartate aminotransferase/SGOT(U/L)	110	7-40
Serum Albumin(g/dL)	3.5	3.4-5.4
Prothrombin(seconds)	12.1	11-13.5
INR	1	0.8-1.1
D- dimer(g/L)	0.5	less than or equal to 0.50
Fibrin degradation products(mcg/mL)	14	less than 10

Pus for culture sent from the echthymatous lesions over the face showed no bacterial growth. Anti-varicella IgM antibodies were positive, and other viral serologies such as HIV, HBsAg, HCV antibodies, and HSV antibodies were negative. The oral prednisolone and MMF were withheld, and the patient was started on intravenous acyclovir 10mg/kg eight hourly within 24 hours of admission and was given for seven days. After one week of treatment, her skin lesions resolved completely, and was discharged in good general condition.

## Discussion

Primary VZV infection in adults is uncommon. Complications of varicella are generally mild, but it can present in severe forms, especially in immunocompromised adults. Severe fatalities are also reported in patients treated with immunosuppressive drugs [9]. The skin lesions in immunosuppressed individuals can present with atypical varicella rash with more lesions, and they can be sick longer than immunocompetent persons. Moreover, in the former, the lesions may continue to erupt for as long as 10 days, may appear on the palms and soles, and may be hemorrhagic [1,3].

These patients are at an increased risk of developing visceral dissemination leading to pneumonitis, hepatitis, encephalitis, acute retinal necrosis syndrome, thrombocytopenia, disseminated intravascular coagulation and hemorrhagic complications ranging from mild febrile purpura to severe and fatal purpura fulminans and “malignant” varicella [1,3]. 

Hemorrhagic varicella, in which a very extensive eruption of hemorrhagic vesicles is accompanied by high fever and severe constitutional symptoms, is rare in an otherwise healthy patient. It is relatively more common in some tropical regions in which malnutrition may be a factor [1]. There have been reports of hemorrhagic varicella in patients with steroid-dependent asthma, chronic liver disease, and nephrotic syndrome [3,4,8]. The clinical presentation of VZV infection in our patients with autoimmune hepatitis and lupus nephritis was atypical. An impaired cellular as well as humoral immunity could be the reason for hemorrhagic varicella in our case [10].

Sharma et al. reported a case of hemorrhagic varicella in a chronic liver disease patient, admitted with discrete vesiculopustular rash and hemorrhagic ulcers in the mouth with frank bleeding from a few lesions., which was managed with aggressive supportive treatment alongside intravenous acyclovir 10 mg/kg eight hourly [8]. Kakinuma et al. reported a case of varicella infection in a patient with leukemia who was on various chemotherapies presented as hemorrhagic bullae., the patient was treated with intravenous infusion of acyclovir, which markedly improved the clinical features [11].

Studies have found that thrombocytopenia is commonly associated with varicella and 30% of patients have platelet count less than 150 x 10^9^/L [12]. Some degree of DIC is frequently seen in immunocompromised patients with varicella [13]. Thrombocytopenia or DIC during acute varicella can be associated with bleeding into skin lesions and surrounding skin or from mucous membranes and visceral hemorrhage. Fatalities are common and result primarily from intractable bleeding leading to intracranial hemorrhage, or pneumonia which is often seen in hemorrhagic varicella. Both our patients had thrombocytopenia but did not show any bleeding manifestations and did not require transfusion or fresh frozen plasma.

Hemorrhagic varicella, resistant to the conventional regimen of acyclovir but responding to continuous infusion of acyclovir has been described [11]. Management of VZV infection in immunocompromised patients is to be considered a matter of great urgency. Immunocompromised patients should avoid contact with patients with VZV infection and if exposed, early administration of VZV immunoglobin and acyclovir is warranted to reduce the morbidity and mortality of the condition [14].

## Conclusions

Hemorrhagic and ecthymatous varicella is a rare, atypical presentation of a commonly seen disease worldwide. The atypical skin lesions seen in immunosuppressed individuals may result in a delay in diagnosis and treatment. Prompt diagnosis and treatment will ameliorate the morbidity and mortality associated with the disease in the immunosuppressed. Physicians should also be aware of the varicella-immune status of their patients on immunosuppressive agents, and, whenever possible, individuals who are seronegative for VZV antibodies should be vaccinated prior to the initiation of immunosuppressive agents. Early diagnosis and prompt treatment become a lifesaving step in such a scenario.
